# Multifunctional Magnetic Catheter Robot with Triaxial Force Sensing Capability for Minimally Invasive Surgery

**DOI:** 10.34133/research.0681

**Published:** 2025-04-24

**Authors:** Shixiong Fu, Shiyuan Dong, Haolan Shen, Zhiqiang Chen, Guoyao Ma, Mingxue Cai, Chenyang Huang, Qianbi Peng, Chenyao Bai, Yuming Dong, Huanhuan Liu, Tianyu Yang, Tiantian Xu

**Affiliations:** ^1^ Guangdong Provincial Key Laboratory of Robotics and Intelligent System, Shenzhen Institutes of Advanced Technology, Chinese Academy of Sciences, Shenzhen, China.; ^2^ University of Chinese Academy of Sciences, Beijing, China.; ^3^ School of Physics and Electronic Engineering, Chongqing Normal University, Chongqing, China.; ^4^ The Academy for Engineering and Technology, Fudan University, Shanghai, China.; ^5^ The Research Centre for Opto-Electronic Engineering and Technology, Shenzhen Institutes of Advanced Technology, Chinese Academy of Sciences, Shenzhen, China.; ^6^ The Key Laboratory of Biomedical Imaging Science and System, Shenzhen Institutes of Advanced Technology, Chinese Academy of Sciences, Shenzhen, China.; ^7^ Shenzhen Key Laboratory of Minimally Invasive Surgical Robotics and System, Shenzhen Institutes of Advanced Technology, Chinese Academy of Sciences, Shenzhen, China.

## Abstract

Magnetic continuum robots offer flexibility and controllability, making them promising for minimally invasive surgery (MIS). However, the clinical application of these robots is relatively limited due to the difficulty of integrating miniaturized triaxial force sensors and their single functionality. This paper proposes a multifunctional magnetic catheter robot with magnetic actuation steering and triaxial force-sensing capabilities. The robot features 3 channels at its tip that integrate multi-segmented magnets, a novel triaxial force sensor, and various functional instruments. The sensor is calibrated, demonstrating high sensitivity and accuracy. The steering characterization of the robot confirms that the catheter tip exhibits effective flexibility and force sensing. Palpation experiments involving various hard lumps are performed on porcine kidney, with results verifying that the robot can reliably detect abnormal hard lumps within tissues. Additionally, palpation experiments in bronchial phantom demonstrate the robot’s imaging and palpation capabilities for lung nodules with an integrated endoscope. Further, the robot, equipped with biopsy forceps, successfully performs palpation and biopsy functions on simulated stomach polyps, demonstrating its capability for effective tissue manipulation. By leveraging force-sensing capabilities and integrating multifunctional instruments, the robot shows potential for expanded applications in MIS, paving the way for important advancements in clinical procedures.

## Introduction

Minimally invasive surgery (MIS) has been widely used in the interventional treatment of various diseases, including cardiovascular and cerebrovascular diseases, digestive system diseases, and lung diseases [1–5]. Compared to traditional open surgeries, MIS offers several key advantages, such as less invasive, quicker postoperative recovery, and lower risk of complications [6–9]. However, conventional catheters commonly used in surgery do not have active steering capability, and the lack of force-sensing capability at the distal end of the catheter is also a important challenge [10,11]. Without precisely sensing the contact force between the catheter tip and surrounding tissues, surgeons risk inadequate or excessive force control, which can lead to puncturing or tissue damage during operation [12]. Therefore, developing new catheters with active steering and force-sensing capabilities can enhance surgical safety and improve the flexibility of catheter operation, thereby helping surgeons better complete interventional surgery [13,14].

Magnetic fields are safe and radiation-free and have been widely used in biomedical applications [15–22]. Furthermore, magnetic continuum robots (MCRs) with active steering capability have a reliable potential for clinical applications [23,24] due to their flexibility and controllability, which is expected to improve traditional interventional procedures. Recently, many research teams have proposed several magnetically controlled interventional surgery robots based on MCRs [23,25]. Nelson and colleagues [26–29] have proposed several variable stiffness magnetic catheter robots for improving MIS. Zhao and colleagues [30,31] proposed a telerobotic neurointervention system with magnetic soft guidewire for stroke treatment. Valdastri and colleagues [32,33] designed a variety of magnetic catheters for endoscopy or vascular intervention. Choi and colleagues [34,35] have developed an electromagnetically controllable micro-robotic intervention system based on magnetic guidewire. Liu and colleagues [36] first proposed a flexible magnetically controlled continuum robot and enlarged the effective working space of the magnetic navigation system, verifying the robot’s flexible steering ability in a set of narrow rings and a kidney phantom. Subsequently, the team also proposed a novel opposite magnetic catheter robot capable of deformation with large angles and high-order curvatures, and completed its kinematic analysis and pose control [37–39]. Xu and colleagues proposed several magnetic soft guidewires and integrated magnetically controlled guidewire robot systems for vascular interventional surgery [40,41], and achieved stable control of the magnetic guidewire by a dual-loop sliding mode control method [42]. While much progress has been achieved in advancing traditional MIS through the aforementioned research, most robots are still limited to the steering functionalities, which consequently restricts their operational versatility and clinical applicability. Recently, several researchers have proposed several MCRs with functional tips to achieve endoscopic imaging and diverse functional applications. For example, Xie and colleagues proposed 2 variable stiffness MCRs with functional tips for intracavitary interventional surgery [43,44], and Misra and colleagues [45] proposed a variable stiffness microgripper for tissue biopsy. Nevertheless, these robots remain constrained by the absence of force-sensing capabilities, which not only impede their progression toward intelligent operation but also restrict their applicability in critical diagnostic procedures such as palpation-based detection of abnormal tissues or tumors. Thus, the advancement of force-sensing technologies plays a crucial role in enhancing the tactile perception capabilities of MCRs during tissue interaction while also ensuring their safe clinical deployment through improved operational control and risk mitigation [46].

Electrical force sensors have historically dominated the application of distal force sensing in catheters due to their early development and availability and their straightforward integration with existing electronic systems. However, these sensors present some drawbacks, such as susceptibility to electromagnetic interference, large size, and limited durability and sensitivity [47,48]. In contrast, optical force sensors, particularly those based on optical fibers, have rapidly gained prominence in the field of medical robotics due to their superior electromagnetic immunity, miniaturization, and high sensitivity [10,49–51]. For example, in cardiac ablation procedures, optical force sensors provide precise force feedback, which is crucial for avoiding tissue damage and improving surgical outcomes. Among optical sensors, force sensors based on fiber Bragg gratings (FBGs) have gradually become one of the mainstream technologies for distal force sensing in catheters due to their immunity to light intensity fluctuations and extremely small size, providing a more reliable solution for force sensing [52,53]. Gan and colleagues [54] designed a highly sensitive biaxial FBG force sensor for vascular interventional surgeries. Li and colleagues [55] designed a tri-axial FBG force sensor for the distal end of a catheter used in cardiac ablation, providing precise force feedback that helps maintain optimal contact with cardiac tissue, thereby reducing the risk of tissue damage and improving procedural safety. Zhang and colleagues [56] proposed a tri-axial FBG force-sensing microneedle for retinal microsurgery. This sensor uses 3 optical fibers arranged at 120° intervals around the circumference of a nitinol tube to sense tri-axial forces, with each fiber containing 2 FBG sensing elements. Liang and colleagues [57] designed a novel tri-axial FBG force sensor for medical robotics, featuring an innovative elastomer structure that utilizes 5 optical fibers and 5 FBGs to achieve high-sensitivity 3-dimensional (3D) force measurements. The aforementioned FBG force sensors employ relatively complex elastomer structures and multiple optical fibers to decouple the tri-axial forces. This complexity makes assembly cumbersome, increasing the risk of alignment errors with the added fibers. Additionally, the sensor sizes are not sufficiently miniaturized, posing challenges for integration into catheters or various medical instruments.

To address the above challenges, we proposed a multifunctional magnetic catheter robot with magnetic actuation steering and triaxial force-sensing capabilities to improve MIS. The entire robot consists of a magnetic multifunctional catheter tip and a flexible catheter body, in which the 3 channels of the catheter tip are respectively integrated with multiple segments of magnets, a novel and compact FBG triaxial force sensor, and a variety of functional instruments. The designed FBG force sensor achieves miniaturization and simplifies the assembly process. The sensor has a diameter of only 1 mm and realizes triaxial force sensing on one optical fiber, which minimizes the assembly error caused by a large number of optical fibers and helps to better integrate with the magnetic catheter. Compared with most existing MCRs, the proposed multifunctional magnetic catheter robot can not only actively steer and navigate under the actuation of an external magnetic field but also obtain real-time force feedback through the FBG force sensor integrated at the tip. In addition, the biopsy forceps and endoscope integrated in the working channel further enhance the operation capability and application range of the magnetic catheter robot, such as palpation of lung nodules, palpation of kidney tumors, and biopsy of stomach polyps in MIS. Therefore, our work provides an effective reference and lays a foundation for the multifunctional integration of MCRs, which helps to improve the environmental perception and multifunctional application capabilities of MCRs.

## Results

### Working principle

MCRs with active steering capabilities hold great promise for clinical applications due to their flexibility and controllability, which can enhance traditional interventional procedures. However, most existing MCRs lack force-sensing capabilities, limiting their potential for intelligent operation, and typically feature only steering functions, which restricts their clinical utility. To address these limitations, it is essential to design new magnetic catheters that integrate advanced sensors and multifunctional tips, enabling MCRs to sense their environment and support multifunctional interventional procedures in conjunction with medical imaging and other medical devices.

**Fig. 1.**
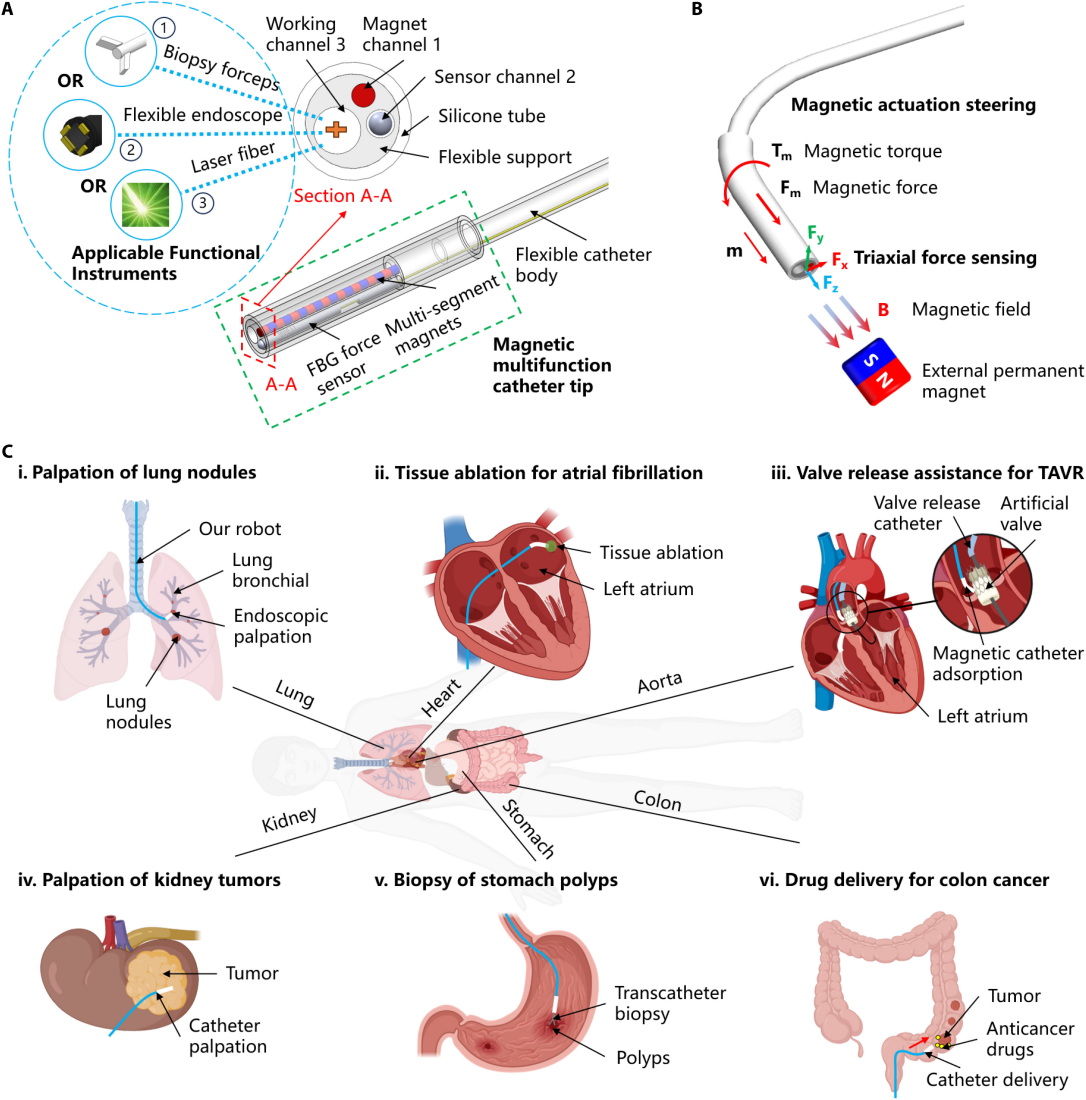
Working principle and application scenarios of multifunctional magnetic catheter robot (MMCR). (A) The overall composition of the MMCR includes an external complete diagram and a cross-sectional diagram A–A. (B) Magnetic actuation steering and triaxial force-sensing principles of the MMCR. (C) Multifunctional application scenarios of MMCR in MIS. (i) Palpation of lung nodules in bronchial. (ii) Tissue ablation for atrial fibrillation. (iii) Valve release assistance for transcatheter aortic valve replacement (TAVR). (iv) Palpation of kidney tumors. (v) Biopsy of stomach polyps. (vi) Drug delivery for colon cancer. (All organ and human model images were created using BioRender and then processed.)

In this work, a multifunctional magnetic catheter robot with magnetic actuation steering and triaxial force-sensing capabilities was proposed to improve MIS (Fig. 1A). The robot comprises a magnetic multifunctional catheter tip and a flexible catheter body. The catheter tip is equipped with 3 channels, each serving a distinct purpose: a magnet channel that integrates a multi-segment magnet for magnetic responsiveness, a sensor channel that houses an indigenously designed FBG triaxial force sensor, and a working channel that accommodates various functional surgical instruments, such as biopsy forceps, a flexible endoscope, and a laser fiber optic. Two important capabilities of the magnetic catheter robot are magnetic actuation steering and triaxial force sensing (Fig. 1B). Actuated by an external magnetic field, the magnetic tip can be flexibly deflected by the magnetic force and torque, which helps to quickly select the target path during intracavitary intervention. The FBG triaxial force sensor provides real-time sensing of contact forces with the surrounding environment, offering force feedback information to support precise robot navigation. When integrated with multiple functional surgical instruments, the proposed magnetic catheter robot has the potential to achieve wider applications of MIS, including lung nodule palpation, tissue ablation for atrial fibrillation, assistance in aortic valve replacement, kidney tumor palpation, stomach polyp biopsy, and drug delivery for colon cancer (Fig. 1C).

### Fabrication and functional integration of magnetic catheter

**Fig. 2.**
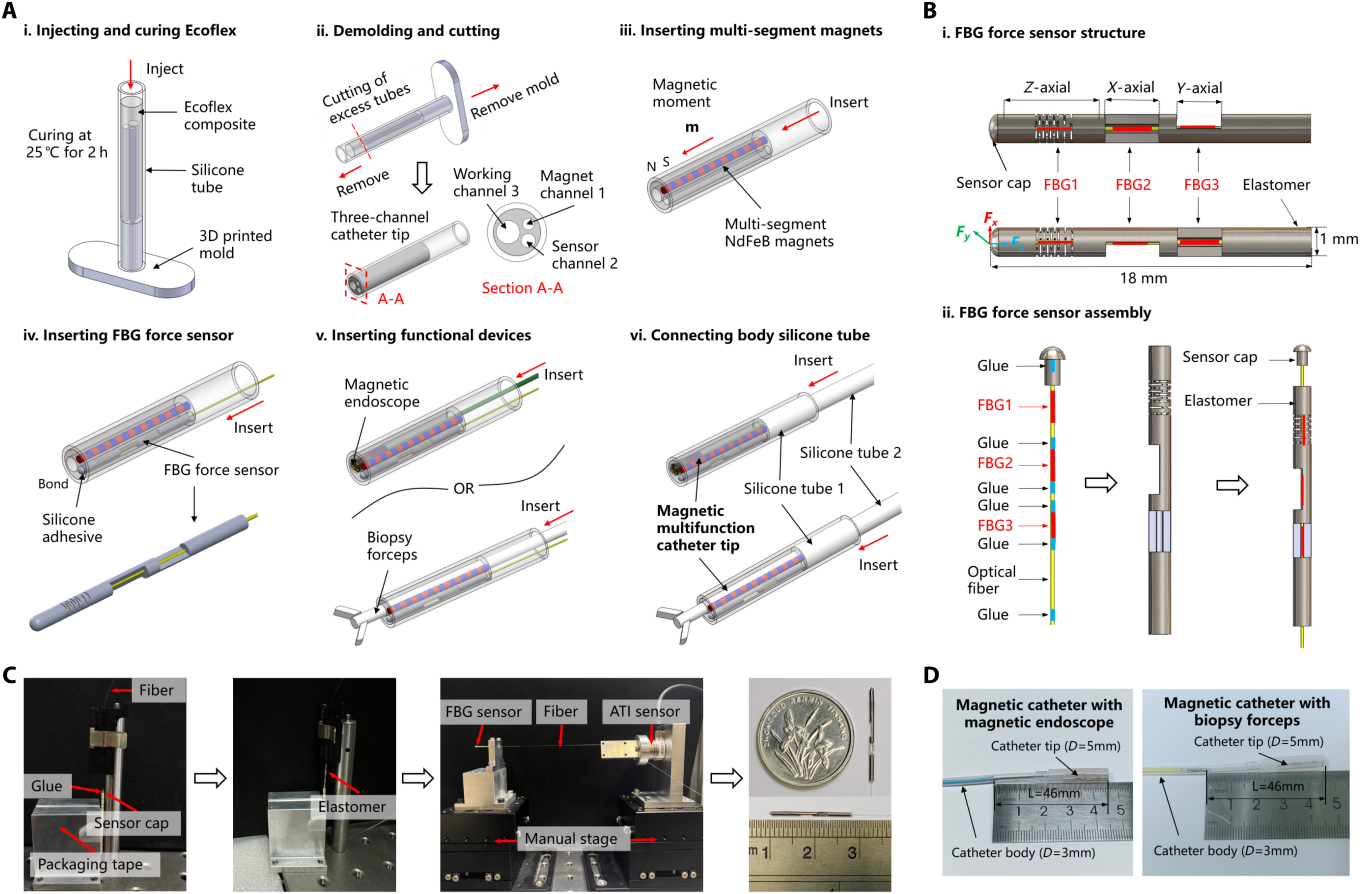
Fabrication process and functional integration of magnetic catheter. (A) Detailed fabrication process. (i) Inject Ecoflex composite into the mold and cure. (ii) Remove the mold and cut the excess tubing to obtain a 3-channel catheter tip. (iii) Insert multi-segment magnets into the catheter tip to obtain magnetic response. (iv) Insert the FBG force sensor into the catheter tip and bond the interface with silicone adhesive. (v) Insert the functional device (e.g., magnetic endoscope and biopsy forceps) into the working channel of the catheter tip. (vi) Connect the body silicone tube to the catheter tip to obtain a complete magnetic catheter. (B) Design and assembly of the FBG triaxial force sensor, which consists of a single optical fiber containing 3 FBGs, a sensor cap, and an elastic structure. (i) Design structure and dimensions of the FBG force sensor. (ii) Schematic diagram of the assembly of the FBG force sensor. (C) Actual assembly process and prototype of the FBG force sensor. (D) Prototypes and dimensions of 2 magnetic catheters with integrated magnetic endoscope and biopsy forceps.

The fabrication process of the magnetic catheter is illustrated in Fig. 2A and consists of the following 6 steps. Step 1 involves injecting and curing Ecoflex. A silicone tube with an inner diameter of 4 mm and an outer diameter of 5 mm is inserted into a 3D-printed mold. A flexible composite of Ecoflex-0030 is prepared, injected into the mold, and cured at 25 °C for 2 h. Step 2 is demolding and trimming. After curing, the 3-channel catheter tip is obtained by removing the mold and trimming the excess tube. The cross-section A–A of the tip comprises a working channel, a magnet channel, and a sensor channel. Step 3 involves inserting multiple magnetic segments. N52-grade NdFeB magnets (1 mm in diameter and 2 mm in length) are inserted into the magnet channel of the catheter tip, with their magnetic moments aligned to enable magnetic steering under an external magnetic field. Step 4 is the insertion of the FBG force sensor. The FBG force sensor is inserted into its designated channel, and the interface between the sensor cap and the silicone tube is bonded with silicone adhesive to ensure stability. Step 5 is the integration of functional devices. Various compatible medical instruments can be inserted into the working channel at the catheter tip, such as a magnetic endoscope with a ring magnet for endoscopic diagnostics or biopsy forceps for tissue sampling. Step 6 involves connecting the main silicone tube. The main silicone tube, with an inner diameter of 2 mm and an outer diameter of 3 mm, is inserted into the tail cavity of the catheter tip, ensuring a secure connection. Upon completion, the magnetic multifunctional catheter robot features a tip with an outer diameter of 5 mm and a main body with an outer diameter of 3 mm, offering triaxial force sensing and magnetic actuation steering capabilities.

A novel FBG triaxial force sensor was presented, comprising a single optical fiber, a sensor cap, and an elastic structure, as illustrated in Fig. 2B. The proposed structure consists of 2 primary components: an elastic body segmented into 3 sections, each specifically responsive to forces in designated directions, and a top tip designed for force application. The integrated elastic structure was divided into 3 regions from top to bottom, which were selectively sensitive to forces in the *Z*, *X*, and *Y* directions, respectively. Each region incorporated an FBG to measure contact forces along its corresponding axis. FBG1 was positioned in a cylindrical cavity and exhibited exclusive sensitivity to forces along the Z direction. To enhance the sensitivity to axial forces while mitigating the influence of lateral elastic strain, rectangular and parallelogram groove beam structures were engraved on the surface. FBG2 and FBG3 were embedded in 2 mutually perpendicular cantilever beams, facilitating effective decoupling of lateral forces along the *X* and *Y* directions. A single-mode optical fiber with an acrylate coating and an overall diameter of 0.25 mm was employed, with each of the 3 FBGs having a length of 2 mm and a reflectivity exceeding 50%. The central wavelengths of the 3 FBGs were 1,545, 1,550, and 1,555 nm, respectively.

After processing all parts of the sensor, they need to be assembled. An assembly platform was designed and built to facilitate sensor assembly, as shown in Fig. 2C. First, the optical fiber and the sensor cap were assembled. The optical fiber was fixed in an optical fiber fixture, while the sensor cap was secured with glue and a support fixture. The optical fiber fixture was then slowly moved to vertically insert the optical fiber into the optical fiber hole, and Loctite 4011 instant adhesive was used for bonding. Next, the optical fiber was bonded to the elastomer components, as depicted in the figure. The designed sensor was fixed in a manual translation stage, and an ATI standard force sensor was mounted on another manual translation stage. The optical fiber was fixed in a fixture connected to the front end of the ATI standard force sensor with glue, and the manual translation stage was adjusted until the ATI standard force sensor reading reaches 0.2 N. Applying pre-tension to the optical fiber in advance helps to avoid the chirp effect and ensures that the optical fiber remained taut within the elastomer, thereby improving linearity. After bonding, the disintegrator separated the optical fiber from the fixture. It was necessary to wait for 24 h after each bonding step to allow the Loctite 4011 adhesive to fully cure. The sensor tip had a diameter of 1 mm and a length of 18 mm.

Finally, 2 magnetic multifunctional catheters were obtained through the above fabrication method, namely, a magnetic catheter with a magnetic endoscope and a magnetic catheter with a biopsy forceps. Both of them had the ability of magnetically driven steering and triaxial force sensing, and the dimensions of the catheter tip were 5 mm in diameter and 46 mm in length, and the outer diameter of the catheter body was 3 mm (Fig. 2D).

### Sensor calibration and magnetic catheter steering characterization

**Fig. 3.**
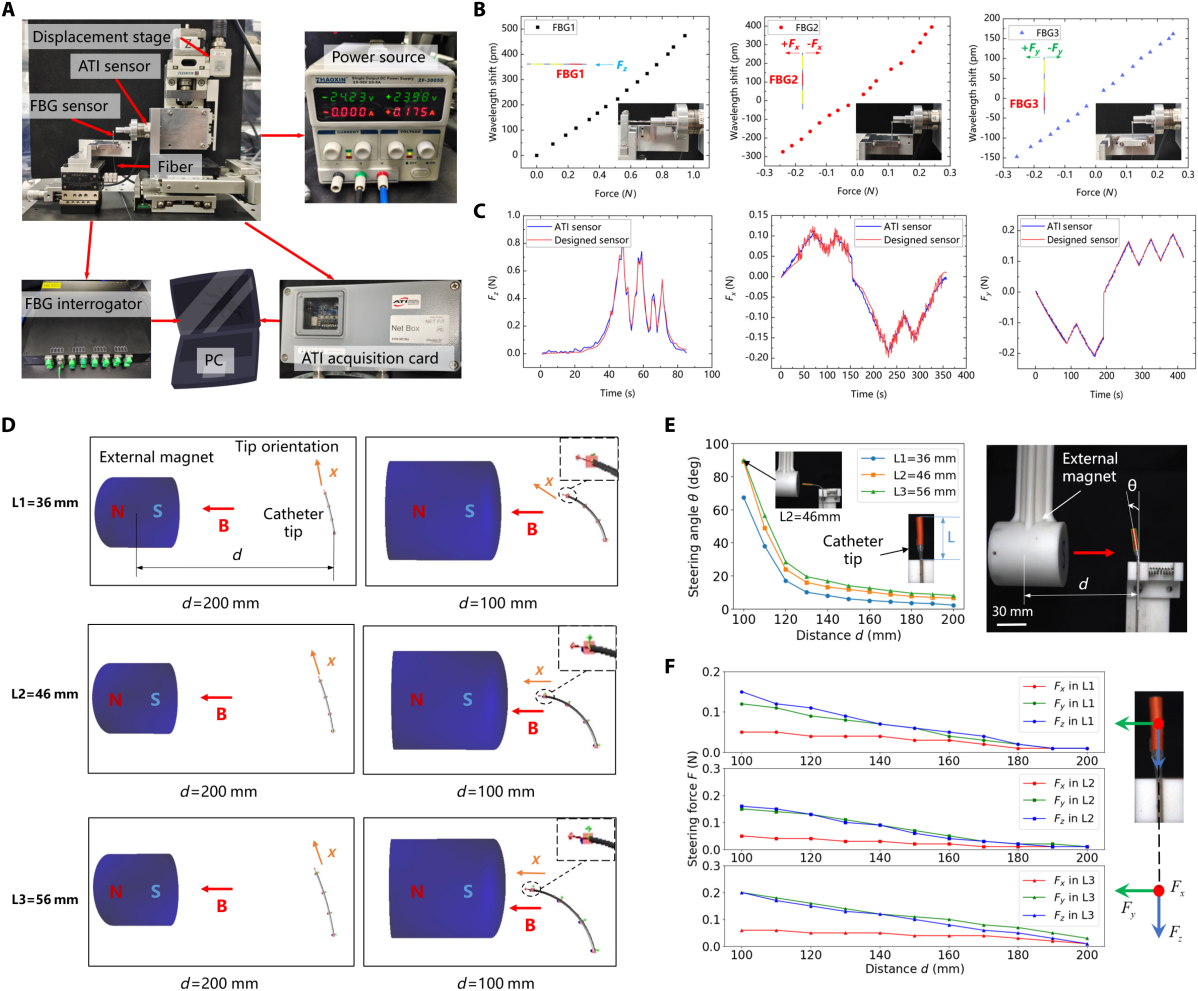
Sensor calibration and magnetic catheter steering characterization. (A) Experimental setup for FBG force sensor calibration. (B) Calibration results of FBG force sensor in 3 axes. (C) Comparison of force measurements of FBG force sensor and ATI sensor in 3 axes. (D) Simulation results of deflection of the magnetic catheter at 3 sets of tip lengths, L1 = 36 mm, L2 = 46 mm, L3 = 56 mm. (E) Characterization results of the steering angle of the magnetic catheter and the working distance at 3 sets of tip lengths. (F) Characterization results of triaxial steering force value on the catheter tip and working distance.

After completing the fabrication and assembly of the FBG force-torque sensor, it was necessary to build an experimental setup to calibrate it using a standard ATI force-torque sensor (Fig. 3A). Fiber optic at the end of the FBG force sensor is fused to a fiber optic patch cable, which is then connected to the FBG interrogator to record the center wavelength drift of the FBG force sensor in real time. The ATI sensor recorded the detected force data in real time, and by moving the displacement stage to let the ATI sensor apply a contact force on the FBG force sensor, and at the same time record the center wavelength drift and the force value of the ATI sensor, and ultimately obtain the results of the FBG force sensor in the 3 axes of *Z*, *X*, and *Y* (Fig. 3B). The calibrated force sensitivity coefficients are *KZ* = 494.86 pm/N, *KX* = 1,338.99 pm/N, and *KY* = 623.35 pm/N, and the designed sensor has high sensitivity.

Dynamic response experiments on the force sensors were conducted to verify the accuracy, hysteresis, and other parameters of the designed force sensors. According to the results of the calibration of the FBG force sensor mentioned above, the ATI sensor was used to continuously apply force to the designed sensor in the 3-axis directions of *Z*, *X*, and *Y*, respectively, and the respective force data were recorded in real time, and finally, a comparison graph of the 2 force measurements was plotted (Fig. 3C). From the graph, it can be seen that the designed force sensor has high accuracy and good hysteresis with root mean square error (RMSE) of 2.08%, 4.81%, and 1.09% in the 3 directions, respectively. Therefore, the designed FBG force sensor has high accuracy in all 3 directions and can accurately measure the contact force values in the subsequent experiments.

After completing the structural design of the magnetic catheter, the deflection simulation could be performed to preliminarily verify the catheter’s steering ability and improve the catheter’s size, thereby shortening the manufacturing cycle of the catheter. Based on the Simulation Open Framework Architecture (SOFA) platform, this paper conducted a deflection simulation of the designed magnetic catheter, selected 3 different catheter tip lengths, namely, L1 = 36 mm, L2 = 46 mm, and L3 = 56 mm, and set the relevant material parameters and dimensions of the catheter, and then designed a cylindrical magnet (with a diameter of 60 mm and a thickness of 60 mm) and imported it into the simulation environment, and generated magnetic field distribution data to calculate the magnetic force and torque on the catheter tip. In the simulation, the S pole of the control magnet was slowly moved from 200 mm away from the initial position of the catheter to 100 mm, and 3 sets of simulation results were obtained (Fig. 3D). It could be seen from the figure that at the same working distance *d*, the longer the catheter tip was, the greater the deformation caused by the magnetic field acting on the tip, that is, the larger the deflection angle, and at a distance of 100 mm, the turning angle of the catheter with a tip length of L1 was close to 70°. In contrast, the catheters corresponding to L2 and L3 could both be close to 90°. If the distance continues to shorten, the catheter could deflect at a larger angle, so the latter 2 groups of catheter lengths could meet actual application needs.

A magnetic catheter was manufactured with only FBG force sensors, without functional devices, to characterize the relationship between the steering angle, steering force, and working distance of the magnetic catheter. Experiments were carried out in turn for 3 groups of tip lengths. The rigid part of the tip was marked with red to facilitate the measurement of the deflection angle with a camera. A cylindrical magnet (60 mm in diameter and 60 mm in thickness) was installed on the end of the UR10 robot through a fixture. The magnet was controlled to slowly approach from 200 mm to 100 mm from the initial position of the catheter. During the process, the working distance, the tip steering angle, and the triaxial steering force value data of the tip were measured and saved, and finally, the change curve between the 3 was drawn (Fig. 3E and F). In Fig. 3E, at a distance of 100 mm, the steering angle of the catheter with a tip length of L1 is 67°, while the catheters corresponding to L2 and L3 can reach 90°, which is consistent with the results of the catheter simulation mentioned above. As can be seen from Fig. 3F, the catheter tip is mainly affected by the steering force in the *Y*–*Z* plane. Hence, the forces on the *Y* and *Z* axes are larger, the *X*-axis force is the smallest, and, as the working distance shortens, the steering forces on each axis gradually increase; at the same time, the longer the tip, the greater the triaxial combined force it is subjected to.

### Ex vivo palpation of simulated tumor lumps in the porcine kidney

**Fig. 4.**
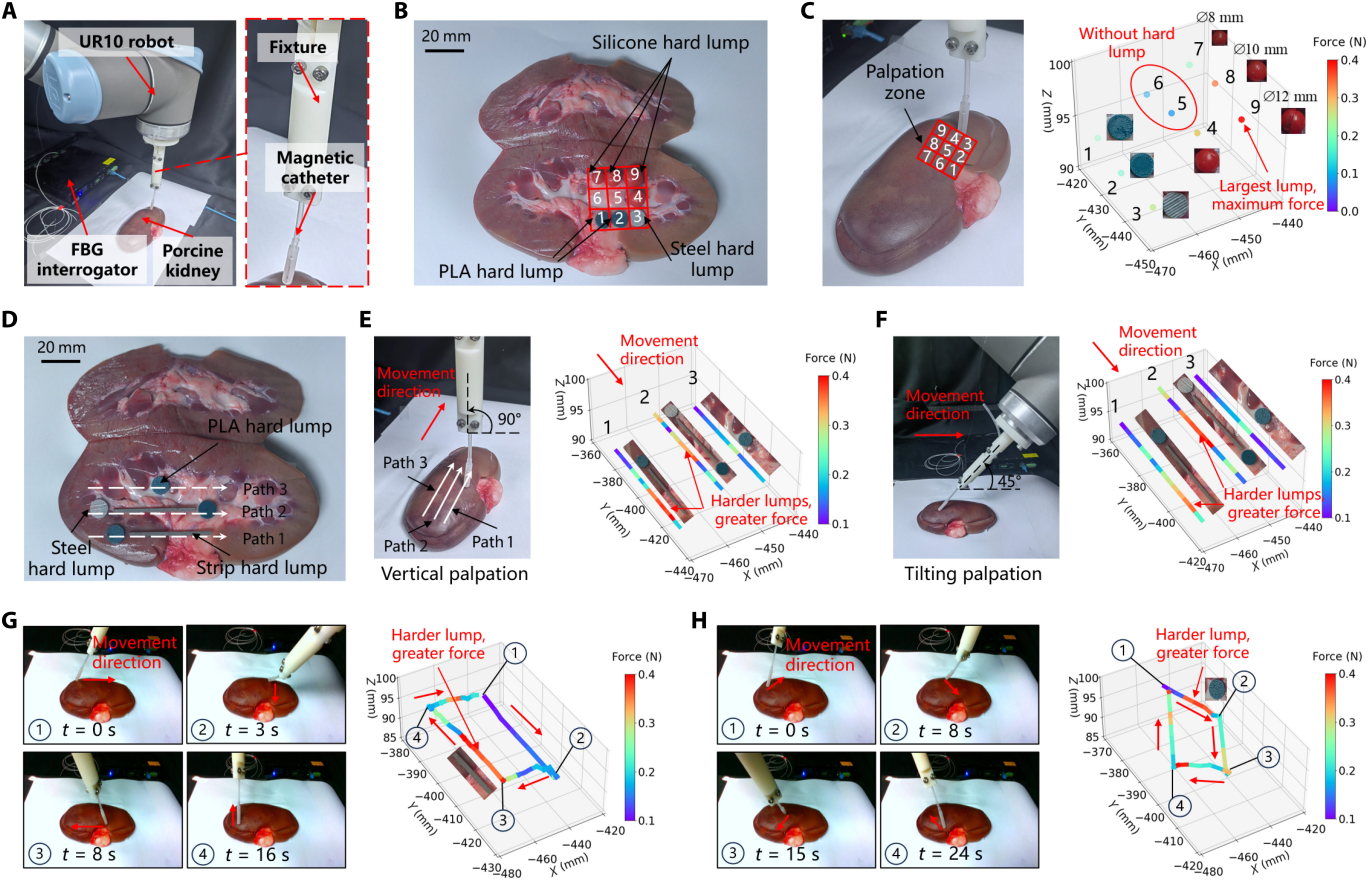
Application of ex vivo palpation of simulated tumor lumps in the porcine kidney. (A) Experimental setup for porcine kidney palpation. (B) Distribution of hard lumps in discrete palpation. (C) Color map of the total force values of discrete palpation points. (D) Distribution of hard lumps in continuous palpation. (E) Total palpation force values for 3 continuous paths with the catheter tip vertically at 90°. (F) Total palpation force values for 3 continuous paths with the catheter tip-tilted at 45°. (G) Palpation force for a rectangular continuous path. (H) Palpation force for a diamond continuous path.

Lesional tissues typically exhibit increased hardness compared to normal tissues. To assess the ability of the designed magnetic catheter to identify internal abnormal tissues during palpation procedures, this study conducted experiments on palpating hard lumps within the porcine kidney. The experimental setup is illustrated in Fig. 4A (details are provided in Materials and Methods), and a prototype magnetic catheter was fabricated with an FBG-based force sensor capable of force measurement. Two sets of palpation experiments were performed: discrete palpation and continuous palpation. In the discrete palpation experiments, silicone hard lumps of varying sizes, along with polylactic acid (PLA) hard lumps and steel hard lumps of different hardnesses, were embedded inside the isolated kidney to simulate diverse lesion scenarios (Fig. 4B). A UR10 robotic arm was utilized to press the magnetic catheter tip into the isolated kidney by a depth of 1 to 2 mm at each of the 9 palpation points. The corresponding force applied at the catheter tip was measured using an FBG interrogator to detect the presence of lesions (Fig. 4C). The results revealed varying force values detected by the FBG force sensor across the 9 positions. At points 5 and 6, located within normal tissue without hard lumps, the measured forces were minimal at 0.08 and 0.09 N, respectively. At points 1 and 2, where PLA hard lumps were present, and at point 3, where steel hard lumps were located, larger and more varied force values of 0.19, 0.17, and 0.25 N were recorded, indicating that force values increased in the presence of anomalies. Furthermore, these force measurements demonstrated distinct variations based on the hardness of the lumps, with greater force values detected for harder objects. This confirmed the ability of the FBG force sensor to differentiate the hardness of lesions during palpation. At points 4, 7, 8, and 9, where silicone lumps of varying diameters were used, the force values ranged from 0.19 to 0.41 N. Larger-diameter silicone lumps produced higher force values, while smaller-diameter silicone lumps resulted in lower force values. All force values for lesions with hard lumps exceeded those recorded for normal tissue without hard lumps, further validating the sensitivity of the force sensor.

Continuous palpation experiments were conducted to better simulate real-world scenarios and eliminate the need for consistent pressure depth during each palpation cycle. Hard lumps and elongated strips were placed inside the isolated kidney to represent abnormal lesions and blood vessels typically encountered during continuous palpation (Fig. 4D). In these experiments, the UR10 robotic arm was programmed to press the magnetic catheter vertically at a 90° angle into the isolated kidney at a depth of 1 to 2 mm, with slight variations due to the uneven surface of the kidney. By controlling the end-effector motion of the UR10 robot, the tip of the magnetic catheter was guided to palpate the kidney surface along 3 distinct paths. The combined force applied to the catheter tip was recorded over the path by the FBG interrogator (Fig. 4E). The results showed that force values detected along the paths with hard lumps and long strips were significantly higher compared to regions without any such anomalies. To further evaluate the effectiveness of multi-angle palpation, continuous palpation experiments were also conducted on the isolated kidney at a 45° tilt, and the resulting color map of force values was obtained from the FBG interrogator (Fig. 4F). The data indicated that the force values measured at sites with anomalies were considerably higher than those measured at normal tissue sites during the 45° tilt. Additionally, 2 extra experiments were carried out along rectangular and diamond paths to test the catheter’s performance during continuous palpation at variable angles (Fig. 4G and H). The results also indicated a significant increase in force for the abnormal region containing the lumps, with harder lumps generating greater force. The dynamic process of the experiments is shown in Movie S1. Through the above discrete and continuous palpation experiments, it was confirmed that the designed magnetic catheter effectively detects tissue abnormalities and distinguishes the regions containing vessels.

### Endoscope-assisted palpation of lung nodules in bronchial phantom

**Fig. 5.**
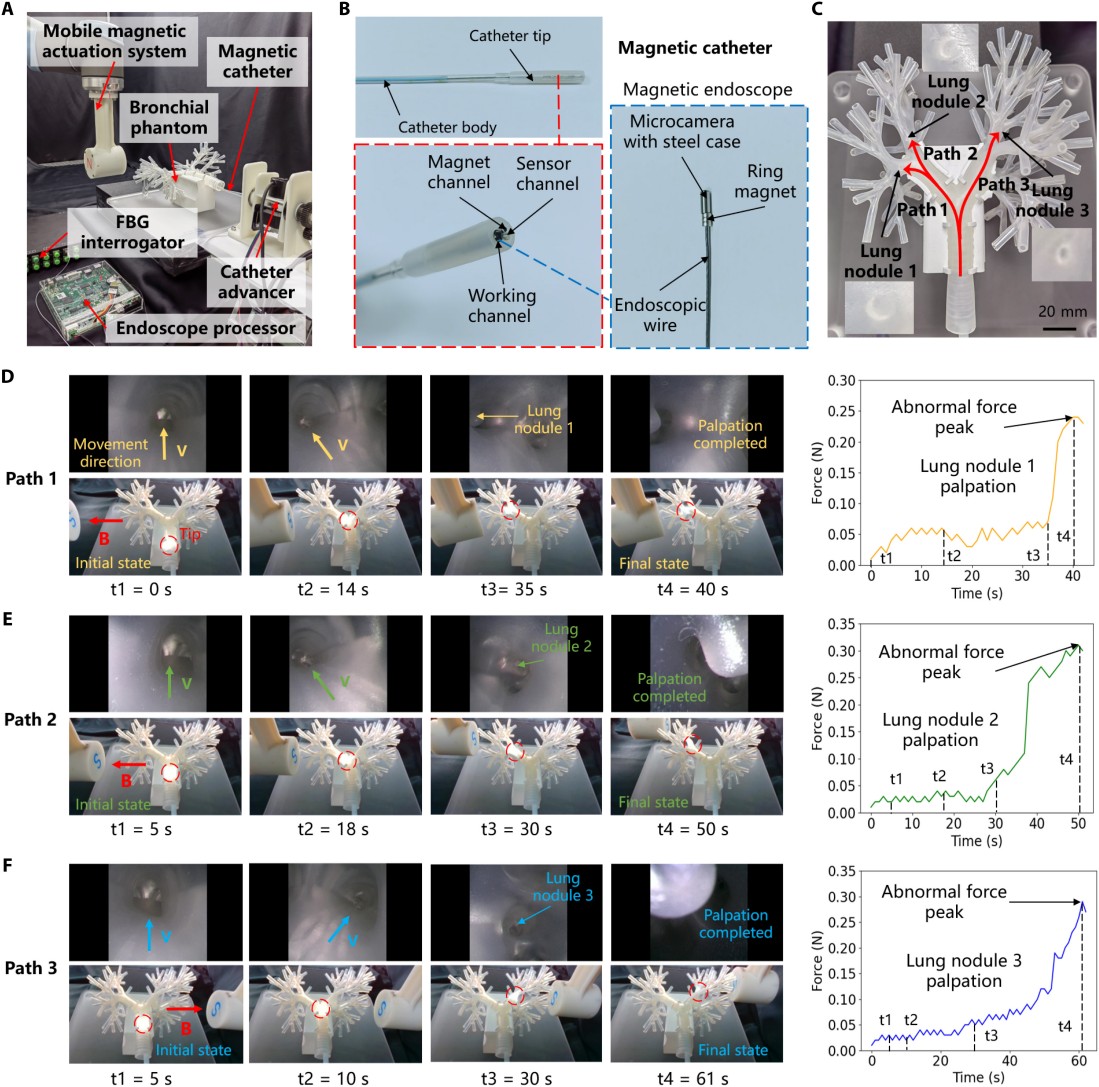
Application of endoscope-assisted palpation of lung nodules in bronchial phantom. (A) Experimental setup for palpation of lung nodules. (B) Magnetic catheter prototype with an integrated magnetic endoscope. (C) Three palpation paths and 3 lung nodules on the bronchial phantom. (D) Palpation process and force change curve of path 1. An abnormal force peak was detected when lung nodule 1 was touched. (E) Palpation process and force change curve of path 2. An abnormal force peak was detected when lung nodule 2 was touched. (F) Palpation process and force change curve of path 3. An abnormal force peak was detected when lung nodule 3 was touched.

To validate the functional application of the proposed magnetic catheter for the diagnosis of lung nodules, the lung nodule palpation experiment was conducted in a bronchial phantom. The experimental setup is depicted in Fig. 5A (details are provided in Materials and Methods). A prototype magnetic catheter integrating a magnetic endoscope and an FBG force sensor was developed (Fig. 5B), providing both endoscopic imaging and force-sensing capabilities. The catheter tip measures 46 mm in length and 5 mm in diameter, while the catheter body has a diameter of 3 mm. Before the experiment, a highly realistic lung bronchial phantom was customized, and 3 palpation paths were designed for lung nodules of varying positions and sizes (Fig. 5C). During the experiment, the magnet position of the mobile magnetic actuation system was remotely controlled using a joystick, which regulated the magnitude and direction of the external magnetic field, thereby actuating the catheter tip to actively bend at the bronchial bifurcation and deflect to the target branch. Simultaneously, the catheter advancer was controlled to provide forward movement for the catheter, thereby smoothly navigating to the lesion area. With the assistance of endoscopic imaging of the magnetic catheter, tissue images inside the bronchial are first acquired, which could identify and distinguish whether the target in the image was a lung nodule or normal tissue (see Fig. S6). After locating the lung nodule, the catheter tip was controlled to move forward to palpate the nodule, and the change in palpation force detected by the FBG interrogator was used to estimate the hardness of the nodule.

During the palpation of path 1, the catheter tip completed the palpation of lung nodule 1 in 40 s, detecting an abnormal force peak of 0.24 N (Fig. 5D). Similarly, the palpation processes for paths 2 and 3 took 50 and 61 s, respectively, with abnormal force peaks of 0.31 and 0.29 N (Fig. 5E and F). The dynamic process of the above experiments is shown in Movie S2. The abnormal force detected by the catheter tip during palpation of the nodules was significantly greater than the force recorded during contact with normal tissue. The palpation results from these 3 paths demonstrate that successful palpation of lung nodules at different positions can be achieved by precisely controlling the deflection and advancement of the magnetic catheter tip. In addition, it should be noted that since the magnetic catheter can exert magnetic actuation steering capabilities, the catheter tip will not directly hit the middle tube wall of the bronchial bifurcation (such as point M in Fig. S7), so the tip will not bend passively. As a result, this experiment verified that the proposed multifunctional magnetic catheter can fuse dual-modal information (image and touch) to efficiently complete the diagnosis of lung nodules.

### Palpation and biopsy of simulated polyps in stomach phantom

**Fig. 6.**
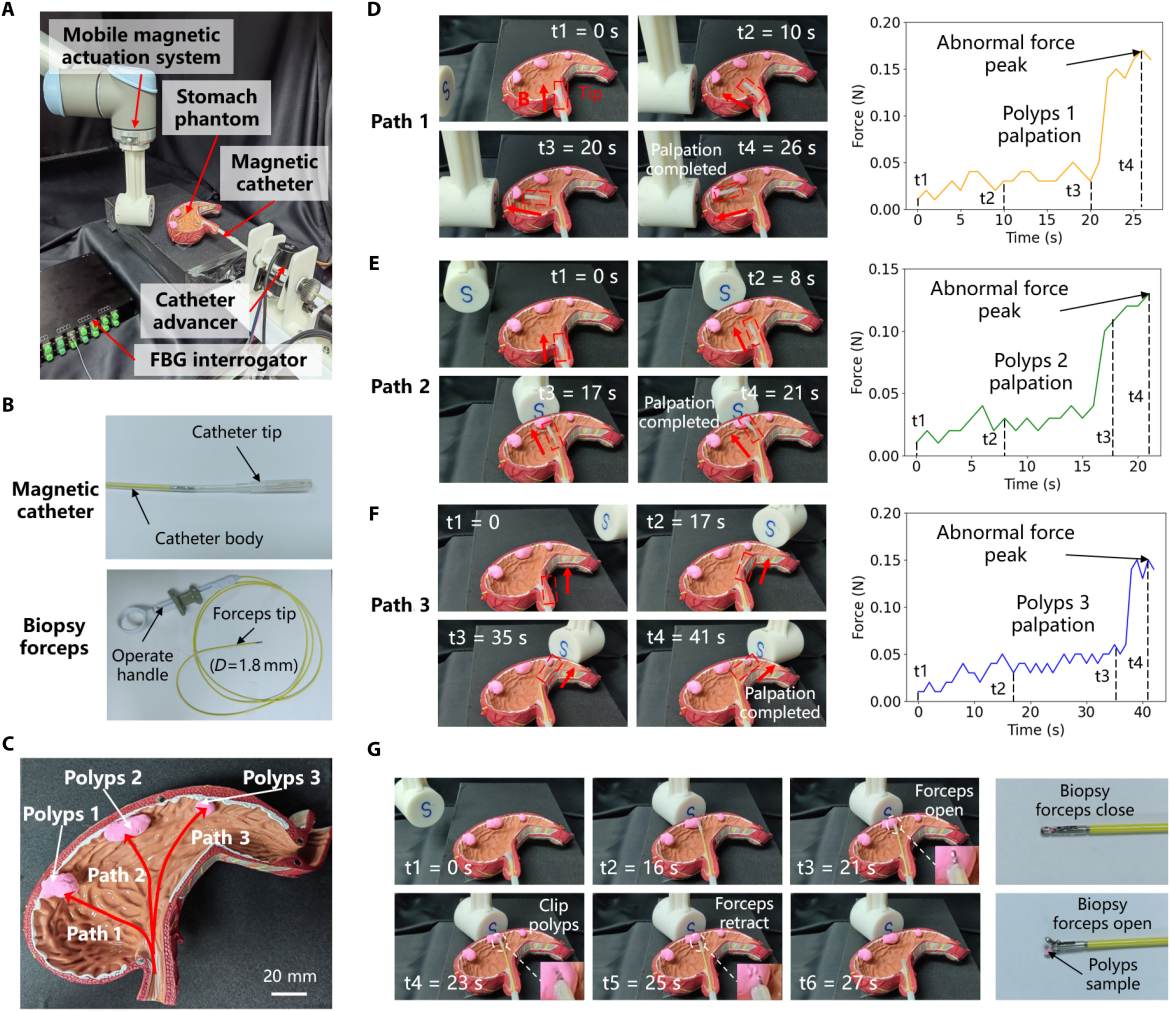
Application of palpation and biopsy of simulated polyps in stomach phantom. (A) Experimental setup for palpation and biopsy of stomach polyps. (B) Magnetic catheter prototype with integrated biopsy forceps. (C) Three palpation paths and 3 simulated polyps on the stomach phantom. (D) Palpation process and force change curve of path 1. An abnormal force peak was detected when polyp 1 was touched. (E) Palpation process and force change curve of path 2. An abnormal force peak was detected when polyp 2 was touched. (F) Palpation process and force change curve of path 3. An abnormal force peak was detected when polyp 3 was touched. (G) Tissue biopsy process of polyp 2. A small polyp sample was taken by controlling the catheter tip and operating the biopsy forceps.

To validate the potential application of the proposed magnetic catheter in stomach disease examination, we conducted palpation and biopsy experiments on simulated polyps using a stomach phantom. The experimental setup is depicted in Fig. 6A, and a magnetic catheter prototype integrating biopsy forceps and an FBG force sensor was developed (Fig. 6B). This catheter offers both tissue biopsy and force-sensing capabilities. The catheter tip measures 46 mm in length and 5 mm in diameter, while the catheter body has a diameter of 3 mm, and the biopsy forceps have a diameter of 1.8 mm. A highly realistic stomach phantom was acquired, and 3 palpation paths were designed for simulated polyps of varying positions and sizes (Fig. 6C). During the experiment, the magnet position in Fig. 6A was remotely controlled using a handle to adjust the magnitude and direction of the external magnetic field, thereby driving the deflection of the catheter tip. Simultaneously, the catheter advancer was controlled to provide forward and backward movement for the catheter, enabling precise navigation during the procedure.

For the 3 palpation paths, we controlled the steering and advancement of the catheter tip to complete the palpation of each polyp and recorded the resultant force changes on the catheter tip over time using the FBG interrogator (Fig. 6D to F). In the palpation experiment of path 1, the catheter tip completed palpation of polyp 1 in 26 s, with a peak abnormal force of 0.17 N detected. Similarly, the palpation processes for paths 2 and 3 took 21 and 41 s, respectively, with peak abnormal forces of 0.13 and 0.15 N. The palpation results from these 3 paths demonstrate that by accurately controlling the deflection direction and forward movement of the magnetic catheter tip, effective palpation of polyps at different positions can be achieved. The abnormal forces detected during contact with the polyps were significantly greater compared to those during contact with normal tissue, highlighting the capability of the catheter to effectively identify abnormalities.

In addition, polyp 2 was selected as the target for tissue biopsy. First, the catheter tip was maneuvered to the vicinity of polyp 2, after which the biopsy forceps were advanced out of the working channel. The tip of the forceps was opened using the handle, and the forceps were then inserted into the polyp to a depth of 1 to 2 mm. The forceps were subsequently closed to grasp the polyp sample, and the biopsy forceps were retracted from the channel to complete the biopsy procedure, successfully obtaining a small polyp sample (Fig. 6G). This experiment thus verified the palpation and biopsy capabilities of the proposed magnetic catheter for stomach polyps. The dynamic process of the above experiments is shown in Movie S3.

## Discussion

Current MCRs face important challenges of insufficient perception and single functions. In this study, we proposed a multifunctional magnetic catheter robot with magnetic actuation steering and triaxial force-sensing capabilities. The 3 channels at the robot’s tip are integrated with multiple segments of magnets, a novel FBG triaxial force sensor, and various functional instruments. The FBG triaxial force sensor was calibrated, and the results showed that the designed sensor has a compact structure, high sensitivity, and accuracy (the performance comparison with other similar sensors is shown in Table S2). At the same time, the steering characterization of the magnetic catheter also confirmed that the catheter tip has good steering ability and force-sensing ability. Discrete palpation and continuous palpation experiments of various hard lumps were carried out on porcine kidneys, and the results verified that the proposed magnetic catheter could stably detect abnormal hard lumps in tissues. For the magnetic catheter integrated with an endoscope, the palpation experiment of 3 lung nodules in a bronchial phantom was conducted to verify the imaging and palpation functions of the magnetic catheter for lung nodules. Subsequently, for the magnetic catheter integrated with biopsy forceps, a simulated polyp palpation and biopsy experiment was carried out in a stomach phantom to verify the palpation and tissue biopsy functions of the magnetic catheter for polyps.

Compared with most existing MCRs, the proposed multifunctional magnetic catheter robot can not only actively steer and navigate under the actuation of an external magnetic field but also obtain real-time force feedback through the FBG force sensor integrated at the tip. Additionally, the integrated biopsy forceps and endoscope within the working channel further enhance the operation capability and application scope of the magnetic catheter. Therefore, our work provides an effective reference and lays a foundation for the multifunctional integration of MCRs, which helps to improve the environmental perception and multifunctional application capabilities of MCRs. In the future, the multifunctional magnetic catheter robot proposed in this article has the potential to be more widely used in MIS by relying on its force perception and integrating more functional instruments.

In future studies, we will further optimize the structure and size of the proposed magnetic catheter, propose a real-time tracking method for the catheter tip, and realize closed-loop motion control of the catheter tip. At the same time, we will also broaden the application scenarios and conduct some in vivo animal experiments to further verify the magnetic catheter’s potential in actual clinical applications.

## Materials and Methods

### Material preparation of magnetic catheter fabrication

The fabrication process of the magnetic catheter is shown in Fig. 2A. Ecoflex is a silicone composite (Ecoflex 00-30, Smooth-On Inc., USA). When used, A glue and B glue are mixed in a 1:1 ratio to fill the catheter tip and provide flexible support for the 3 channels. The silicone tube used in the catheter tip has an inner diameter of 4 mm and an outer diameter of 5 mm, and the size of the main silicone tube is an inner diameter of 2 mm and an outer diameter of 3 mm. The small magnet used in the magnet channel is an N52-grade NdFeB cylindrical magnet (size is 1 mm in diameter and 2 mm in length). The biopsy forceps (Changmei Medical Devices Co. Ltd., China) used in the working channel of the magnetic catheter have a diameter of 1.8 mm, and the magnetic endoscope is a ring magnet (size is 0.6 mm in inner diameter, 2 mm in outer diameter, and 2 mm in length) passed through the endoscope line and reached the tip. The endoscope tip is covered with a stainless steel sleeve to protect the micro camera (model: OVM6946, Haijian Medical Co. Ltd., China), which is a common miniature camera module for medical use and is a core component of medical bronchoscopes and urological scopes. The designed FBG triaxial force sensor mainly includes optical fiber and elastomer. The optical fiber with 3 FBG (Yiqingchen Co. Ltd., China) was manufactured by femtosecond processing technology to ensure optimal sensitivity and precision. The elastomer (Xinpeng Co. Ltd., China) was manufactured by 304 stainless steel, which was selected for its biocompatibility and mechanical stability.

### Magnetic actuation model

Among the methods for describing the magnetic field of permanent magnets, the dipole model is often chosen due to its computational simplicity [9]. In our experiments, the external permanent magnet (EPM) can be approximated as a point source, and therefore, it can be reduced to a magnetic dipole model in a nonuniform field. The magnetic field ***B*** produced by a dipole source on a small magnet inside the tip of a magnetic catheter can be expressed as

$$B=\frac{\mu_{0}}{4\pi {\left\|p\right\|}^{3}}\left(\frac{3pp^{T}}{{\left\|p\right\|}^{2}}-I\right)M$$ (1)

where $\mu_{0}=4\pi \times 10^{-7}\;T\cdot m\cdot A^{-1}$ is the vacuum permeability, p is the distance vector from the center of the external magnet to the magnetic tip ($p=P_{t}-P_{m}$), $I\in {\mathbb{R}}^{3\times 3}$ is an identity matrix, and M is the magnetic dipole moment of the EPM.

The external magnetic field can exert a magnetic force $F_{m}$ and a magnetic torque $T_{m}$ on the internal magnet at the tip of the magnetic catheter, which together actuate the catheter tip to an arbitrarily desired deflection, both of which can be expressed as

$$F_{m}=\left(m\cdot \nabla \right)B$$ (2)

$$T_{m}=m\times B$$ (3)

where m is the magnetic moment of the internal magnet at the tip of the magnetic catheter. ∇ is the gradient operator.

### Principle and simulation of FBG force sensor

The force-sensing principle of the designed FBG triaxial force sensor is shown in Fig. S1. When incident light passes through the grating area where the FBG is located, only the light that meets a specific wavelength is reflected, and the other light is transmitted. This reflected light of a specific wavelength is called the Bragg center wavelength. The Bragg center wavelength is shifted when an external force is applied to the optical fiber to cause strain at a constant temperature. The relationship between wavelength shift and strain can be expressed as follows:

$$\Delta \lambda =\left(1-\rho_{e}\right)\lambda \Delta \varepsilon$$ (4)

where Δλ is the Bragg center wavelength offset and $\rho_{e}$ is the elasto-optical coefficient of the optical fiber. λ is the Bragg center wavelength. Δε is the strain generated. Also due to:

$$\Delta \varepsilon =\frac{\sigma }{E}=\frac{F}{\mathrm{AE}}$$ (5)

where is σ the stress. E is the modulus of elasticity. F is the applied directional force. *A* is the cross-sectional area of the fiber. Therefore, the relationship between the center wavelength offset Δλ and the force F can be written as:

$$\Delta \lambda =\frac{\left(1-\rho_{e}\right)\lambda }{\mathrm{AE}}F=\mathrm{kF}$$ (6)

where is k a constant. From this, it is deduced that the central wavelength offset Δλ varies linearly with respect to the force F. In this sensor structure, since each FBG is only sensitive to the force in a specific direction, the relationship of spatial force to the 3 FBGs can be written as:

$$\Delta \lambda_{1}=k_{Z}F_{Z}$$ (7)

$$\Delta \lambda_{2}=k_{X}F_{X}$$ (8)

$$\Delta \lambda_{3}=k_{Y}F_{Y}$$ (9)

where $\Delta \lambda_{1},\Delta \lambda_{2},\Delta \lambda_{3}$ are the wavelength offsets of FBG1, FBG2, and FBG3, respectively. $k_{Z},k_{X},k_{Y}$ are the force sensitivity coefficients when subjected to axial force and transverse force. $F_{Z},F_{X},F_{Y}$ are the axial force and transverse force.

Finite element simulation analysis of the designed force sensor can predict its performance in advance. The finite element model of the sensor was established in simulation, and the material parameters of the sensor were applied to the model (see Table S1). The elastomer of the sensor was made of 304 stainless steel with good biocompatibility. After meshing, a static force load of 0 to 1 N with an interval of 0.2 N was applied in the *Z* direction, a static force load of −1 to 1 N with an interval of 0.4 N was applied in the *X* direction, and a static force load of −1 to 1 N with an interval of 0.2 N was applied in the *Y* direction. After calling the static structural field module for solution and calculation, the simulation results of the sensor in the 3-axis directions were obtained (as shown in Fig. S2).

Figure S2A to C shows the strain of the force sensor when the sensor applies different *Z*-direction axial forces, and the strain of the sensor and the strain of the optical fiber when an axial force of 1 N is applied; Fig. S2D to F shows the strain of the force sensor when the sensor applies different *X*-direction lateral forces, and the strain of the sensor and the strain of the optical fiber when a 1-N *X*-direction lateral force is applied; Fig. S2G to I shows the strain of the force sensor when the sensor applies different *Y*-direction lateral forces, and the strain of the sensor and the strain of the optical fiber when a 1-N *Y*-direction lateral force is applied. It can be seen that the 3 FBGs are only sensitive to forces in specific directions. The sensor simulation results show that there is a linear relationship between the strain and the axial force in FBG1, a linear relationship between the strain and the lateral force in FBG2, and a linear relationship between the strain and the lateral force in FBG3. The simulated axial sensitivity of the sensor is 271.9 μϵ/N, and the lateral sensitivities are 1,400 μϵ/N and 1,920 μϵ/N, respectively. The corresponding resolutions are 3.68, 0.71, and 0.52 mN, respectively.

### Experimental setup for FBG force sensor calibration

The experimental setup mainly consisted of the designed FBG force sensor, an FBG interrogator (SA-10002454, CASSTK Co. Ltd., China; sampling rate: 100 Hz; resolution: 1 pm) used to record the center wavelength drift of the FBG force sensor, an ATI 6-axis force-torque sensor (Nano17 SI-12-0.12, NC Inc., USA) used to calibrate the FBG sensor, an ATI acquisition card (9105-NETBA, ATI Inc., USA) used to record the detected force data from the ATI sensor, a displacement stage (KOHZU Inc., Japan; resolution: 1 μm) used to let the ATI sensor apply a contact force on the FBG force sensor, a power source, and a PC. Before work, the FBG sensor is connected to the FBG demodulator, which communicates with the PC via a network cable, while the ATI sensor is powered by a power supply, which is connected to the ATI acquisition card and further connected to the PC. All data are finally acquired through the software interface on the PC.

### Preparation of kidney palpation

The experimental setup for the kidney palpation studies included a UR10 robotic arm, magnetic catheters integrated with FBG force sensors, FBG interrogator, 3D-printed fixtures, freshly isolated ex vivo porcine kidneys, and a range of hard lumps (Fig. 4A). The fixture secured the magnetic catheter’s silicone tube, allowing a 60-mm flexible tip segment for free movement and sufficient rigidity for effective palpation force detection, based on the catheter tip lengths used in other palpation experiments and kidney palpation needs. Various hard lumps and strips of different materials and sizes were embedded within the kidneys to simulate pathological tissues and vessels (Fig. 4B and D). These lumps and strips were manufactured using 3D printing and machining, including red silica spheres with diameters of 8, 10, and 12 mm, PLA hard lumps with a diameter of 10 mm, steel hard lumps with a diameter of 10 mm, and strip hard lumps with a length of 40 mm. During the experiments, the UR10 robotic arm’s end movement was controlled using a joystick, which in turn guided the clamped magnetic catheter tip to palpate discrete points and continuous paths on the kidney surface. The force values detected during the palpation process were recorded in real time by an FBG interrogator (SA-10002454, CASSTK Co. Ltd., China).

### Experimental setup for bronchial and stomach palpation

The experimental device used for palpation in the bronchial phantom is shown in Fig. 5A, which specifically includes a magnetic catheter, a mobile magnetic actuation system, a catheter advancer, a bronchial phantom, an FBG interrogator, and an endoscope processor. The mobile magnetic actuation system (details are shown in Fig. S3) includes an N52-grade, 60-mm-diameter, 60-mm-thick cylindrical NdFeB magnet (Jiangpeng Magnetic Materials Co. Ltd., China), a 3D-printed magnet connector, and a UR10 robot (Universal Robots, Denmark). The maximum magnetic field strength of the external magnet is 650 mT. The end motion of the UR10 robot is controlled to change the pose of the permanent magnet so that the magnetic catheter can be deflected to the desired state under the actuation of an external magnetic field. The catheter advancer includes a linear propulsion module and a rotary motion module, which has 2 degrees of freedom and can realize the advancement, retraction, and rotation operations of the catheter. Powered by two 42-stepper motors, the advancer can offer an intervention speed range of 0 to 15 mm/s. The catheter advancer can accommodate different devices with diameters ranging from 2 to 6 mm, which can expand the application scope of the advancer. The detailed structure of the catheter advancer is illustrated in Figs. S4 and S5. The bronchial phantom was made of soft silicone material by 3D printing technology (TRANDOMED Co. Ltd., China) with reference to the size of human lungs. The inner diameter of the tube ranged from 3 to 20 mm, and 3 lung nodules were reserved in the tube. The FBG interrogator (SA-10002454, CASSTK Co. Ltd., China) was used to send and receive optical signals to the optical fiber and decouple the force on the FBG sensor into wavelength drift. The endoscope processor (OK-ES201-V4, JoinHope Image Technologyg Co. Ltd., China) was used to connect the endoscope line and the data acquisition card to obtain images and restore details and finally transmit them to the computer terminal.

## Data Availability

All data needed to evaluate the results in the paper are present in the paper and/or the Supplementary Materials. Additional data related to this paper may be requested from the authors.

## References

[1] Dewire J, Calkins H. Update on atrial fibrillation catheter ablation technologies and techniques. Nat Rev Cardiol. 2013;10(10):599–612. 23979215 10.1038/nrcardio.2013.121

[2] Shi C, Luo X, Qi P, Li T, Song S, Najdovski Z, Fukuda T, Ren H. Shape sensing techniques for continuum robots in minimally invasive surgery: A survey. IEEE Trans Biomed Eng. 2016;64(8):1665–1678. 27810796 10.1109/TBME.2016.2622361

[3] Rafii-Tari H, Payne CJ, Yang GZ. Current and emerging robot-assisted endovascular catheterization technologies: A review. Ann Biomed Eng. 2014;42(4):697–715. 24281653 10.1007/s10439-013-0946-8

[4] Yang GZ, Bellingham J, Dupont PE, Fischer P, Floridi L, Full R, Jacobstein N, Kumar V, McNutt M, Merrifield R, et al. The grand challenges of science robotics. Sci Robot. 2018;3(14):eaar7650. 33141701 10.1126/scirobotics.aar7650

[5] Cianchetti M, Laschi C, Menciassi A, Dario P. Biomedical applications of soft robotics. Nat Rev Mater. 2018;3:143–153.

[6] Polygerinos P, Zbyszewski D, Schaeffter T, Razavi R, Seneviratne LD, Althoefer K. MRI-compatible fiber-optic force sensors for catheterization procedures. IEEE Sensors J. 2010;10(10):1598–1608.

[7] Duan W, Akinyemi T, Du W, Ma J, Chen X, Wang F, Omisore O, Luo J, Wang H, Wang L. Technical and clinical progress on robot-assisted endovascular interventions: A review. Micromachines. 2023;14(1):197. 36677258 10.3390/mi14010197 PMC9864595

[8] Roesthuis RJ, Misra S. Steering of multisegment continuum manipulators using rigid-link modeling and FBG-based shape sensing. IEEE Trans Robot. 2016;32(2):372–382.

[9] Guo J, Shi C, Ren H. Ultrasound-assisted guidance with force cues for intravascular interventions. IEEE Trans Autom Sci Eng. 2018;16(1):253–260.

[10] Taffoni F, Formica D, Saccomandi P, Pino GD, Schena E. Optical fiber-based MR compatible sensors for medical applications: An overview. Sensors. 2013;13(10):14105–14120. 24145918 10.3390/s131014105 PMC3859111

[11] Kesner SB, Howe RD. Design principles for rapid prototyping forces sensors using 3-D printing. IEEE/ASME Trans Mechatron. 2011;16(5):866–870. 10.1109/TMECH.2011.2160353 PMC3160640 21874102

[12] Shah D, Lambert H, Langenkamp A, Vanenkov Y, Leo G, Gentil-Baron P, Walpoth B. Catheter tip force required for mechanical perforation of porcine cardiac chambers. Europace. 2011;13(2):277–283. 21084361 10.1093/europace/euq403

[13] Polygerinos P, Seneviratne LD, Razavi R, Schaeffter T, Althoefer K. Triaxial catheter-tip force sensor for MRI-guided cardiac procedures. IEEE/ASME Trans Mechatron. 2012;18(1):386–396.

[14] Shi C, Li T, Ren H. A millinewton resolution fiber Bragg grating-based catheter two dimensional distal force sensor for cardiac catheterization. IEEE Sensors J. 2017;18(4):1539–1546.

[15] Xu T, Hwang G, Andreff N, Regnier S. Planar path following of 3-D steering scaled-up helical microswimmers. IEEE Trans Robot. 2015;31(1):117–127.

[16] Wu X, Liu J, Huang C, Su M, Xu T. 3-D path following of helical microswimmers with an adaptive orientation compensation model. IEEE Trans Autom Sci Eng. 2019;17(2):823–832.

[17] Yang Z, Zhang L. Magnetic actuation systems for miniature robots: A review. Adv Intell Syst. 2020;2(9):2000082.

[18] Xu S, Liu J, Yang C, Wu X, Xu T. A learning-based stable servo control strategy using broad learning system applied for microrobotic control. IEEE Trans Cybern. 2021;52(12):13727–13737. 10.1109/TCYB.2021.3121080 34714762

[19] Xu T, Hao Z, Huang C, Yu J, Zhang L, Wu X. Multimodal locomotion control of needle-like microrobots assembled by ferromagnetic nanoparticles. IEEE/ASME Trans Mechatron. 2022;27(6):4327–4338.

[20] Xu T, Huang C, Lai Z, Wu X. Independent control strategy of multiple magnetic flexible millirobots for position control and path following. IEEE Trans Robot. 2022;38(5):2875–2887.

[21] Liu J, Wu X, Huang C, Manamanchaiyaporn L, Shang W, Yan X, Xu T. 3-D autonomous manipulation system of helical microswimmers with online compensation update. IEEE Trans Autom Sci Eng. 2020;18(3):1380–1391.

[22] Cai M, Qi Z, Cao Y, Liu X, Wu X, Xu T, Zhang L. Performance-guided rotating magnetic field control in large workspaces with reconfigurable electromagnetic actuation system. IEEE Trans Robot. 2024;40:4117–4131.

[23] Yang Z, Yang H, Cao Y, Cui Y, Zhang L. Magnetically actuated continuum medical robots: A review. Adv Intell Syst. 2023;5(6):2200416.

[24] Huo Y, Yang L, Xu T, Sun D. Design, control, and clinical applications of magnetic actuation systems: Challenges and opportunities. Adv Intell Syst. 2024;7(3):2400403.

[25] Hwang J, Jy K, Choi H. A review of magnetic actuation systems and magnetically actuated guidewire-and catheter-based microrobots for vascular interventions. Intell Serv Robot. 2020;13:1–14.

[26] Chautems C, Tonazzini A, Floreano D, Nelson BJ. A variable stiffness catheter controlled with an external magnetic field. Paper presented at: 2017 IEEE/RSJ International Conference on Intelligent Robots and Systems (IROS); 2017; Vancouver, BC, Canada.

[27] Edelmann J, Petruska AJ, Nelson BJ. Magnetic control of continuum devices. Int J Robot Res. 2017;36(1):68–85.

[28] Chautems C, Tonazzini A, Boehler Q, Jeong SH, Floreano D, Nelson BJ. Magnetic continuum device with variable stiffness for minimally invasive surgery. Adv Intell Syst. 2020;2(6):1900086.

[29] Gervasoni S, Lussi J, Viviani S, Boehler Q, Ochsenbein N, Moehrlen U, Nelson BJ. Magnetically assisted robotic fetal surgery for the treatment of spina bifida. IEEE Trans Med Robot Bionics. 2022;4(1):85–93.

[30] Kim Y, Parada GA, Liu S, Zhao X. Ferromagnetic soft continuum robots. Sci Robot. 2019;4(33):eaax7329. 33137788 10.1126/scirobotics.aax7329

[31] Kim Y, Genevriere E, Harker P, Choe J, Balicki M, Regenhardt RW, Vranic JE, Dmytriw AA, Patel AB, Zhai X. Telerobotic neurovascular interventions with magnetic manipulation. Sci Robot. 2022;7(65):eabg9907. 35417201 10.1126/scirobotics.abg9907 PMC9254892

[32] Norton JC, Slawinski PR, Lay HS, Martin JW, Cox BF, Cummins G, Desmulliez MPY, Clutton RE, Obstein KL, Cochran S, et al. Intelligent magnetic manipulation for gastrointestinal ultrasound. Sci Robot. 2019;4(31):eaav7725. 31380501 10.1126/scirobotics.aav7725 PMC6677276

[33] Pittiglio G, Lloyd P, da Veiga T, Onaizah O, Pompili C, Chandler JH, Valdastri P. Patient-specific magnetic catheters for atraumatic autonomous endoscopy. Soft Robot. 2022;9(6):1120–1133. 35312350 10.1089/soro.2021.0090 PMC9805888

[34] Jeon S, Hoshiar AK, Kim K, Lee S, Kim E, Lee S, Kim JY, Nelson BJ, Cha HJ, Yi BJ, et al. A magnetically controlled soft microrobot steering a guidewire in a three-dimensional phantom vascular network. Soft Robot. 2019;6(1):54–68. 30312145 10.1089/soro.2018.0019 PMC6386781

[35] Hwang J, Jeon S, Kim B, Kim JY, Jin C, Yeon A, Yi BJ, Yoon CH, Park HJ, Pané S, et al. An electromagnetically controllable microrobotic interventional system for targeted, real-time cardiovascular intervention. Adv Healthc Mater. 2022;11(11):e2102529. 35137568 10.1002/adhm.202102529

[36] Lin D, Wang J, Jiao N, Wang Z, Liu L. A flexible magnetically controlled continuum robot steering in the enlarged effective workspace with constraints for retrograde intrarenal surgery. Adv Intell Syst. 2021;3(10):2000211.

[37] Lin D, Jiao N, Wang Z, Liu L. A magnetic continuum robot with multi-mode control using opposite-magnetized magnets. IEEE Robot Autom Lett. 2021;6(2):2485–2492.

[38] Lin D, Li N, Jiao N, Wang Z, Liu L. Kinematic analysis of multi-section opposite magnetic catheter robots with solution multiplicity. IEEE Trans Autom Sci Eng. 2022;21(1):123–134.

[39] Lin D, Chen W, He K, Jiao N, Wang Z, Liu L. Position and orientation control of multi section magnetic soft microcatheters. IEEE/ASME Transactions on Mechatronics. 2022;28(2):907–918.

[40] Zhang S, Yin M, Lai Z, Huang C, Wang C, Shang W, Wu X, Zhang Y, Xu T. Design and characteristics of 3D magnetically steerable guidewire system for minimally invasive surgery. IEEE Robot Autom Lett. 2022;7(2):4040–4046.

[41] Fu S, Chen B, Li D, Han J, Xu S, Wang S, Huang C, Qiu M, Cheng S, Wu X, et al. A magnetically controlled guidewire robot system with steering and propulsion capabilities for vascular interventional surgery. Adv Intell Syst. 2023;5(11):2300267.

[42] Xu S, Chen B, Li D, Fu S, Wu X, du S, Xu T. An automatic magnetically robotic system using a double-loop stable control method for guidewire steering. IEEE/ASME Trans Mechatron. 2024.

[43] Mao L, Yang P, Tian C, Shen X, Wang F, Zhang H, Meng X, Xie H. Magnetic steering continuum robot for transluminal procedures with programmable shape and functionalities. Nat Commun. 2024;15(1):3759. 38704384 10.1038/s41467-024-48058-x PMC11069526

[44] Yang P, Mao L, Tian C, Meng X, Xie H. A cooperative and multifunctional magnetic continuum robot for noninteractive access, dexterous navigation, and versatile manipulation. Adv Funct Mater. 2025;35(2):2412543.

[45] Thomas TL, Bos J, Huaroto JJ, Kalpathy Venkiteswaran V, Misra S. A magnetically actuated variable stiffness manipulator based on deployable shape memory polymer springs. Adv Intell Syst. 2024;6(2):2200465.

[46] Huang Y, Ma R, Liu H. A hybrid force-magnetic control scheme for flexible medical device steering. Mechatronics. 2023;95: Article 103072.

[47] Allen H, Ramzan K, Knutti J, Withers S. A novel ultra-miniature catheter tip pressure sensor fabricated using silicon and glass thinning techniques. MRS Proc. 2001;681:I7.4.

[48] Esashi M, Komatsu H, Matsuo T, Takahashi M, Takish T, Imabayashi K, Ozawa H. Fabrication of catheter-tip and sidewall miniature pressure sensors. IEEE Trans Electron Dev. 1982;29(1):57–63.

[49] Menciassi A, Eisinberg A, Carrozza MC, Dario P. Force sensing microinstrument for measuring tissue properties and pulse in microsurgery. IEEE/ASME Trans Mechatron. 2003;8(1):10–17.

[50] Gao A, Zhou Y, Cao L, Wang Z, Liu H. Fiber Bragg grating-based triaxial force sensor with parallel flexure hinges. IEEE Trans Ind Electron. 2018;65(10):8215–8223.

[51] Dong S, Liu Z, Lou Y, Luo D, Wu J, Yang B, Liu H, Yang T, Dong Y. A high-precision miniature triaxial FBG force sensor for detecting tissue anomalies. J Lightwave Technol. 2024;42(17):6143–6152.

[52] Li T, Shi C, Tan Y, Li R, Zhou Z, Ren H. A diaphragm type fiber Bragg grating vibration sensor based on transverse property of optical fiber with temperature compensation. IEEE Sensors J. 2016;17(4):1021–1029.

[53] Iordachita I, Sun Z, Balicki M, Kang JU, Phee SJ, Handa J, Gehlbach P, Taylor R. A sub-millimetric, 0.25 mN resolution fully integrated fiber-optic force-sensing tool for retinal microsurgery. Int J Comput Assist Radiol Surg. 2009;4(4):383–390. 20033585 10.1007/s11548-009-0301-6 PMC2801926

[54] Gan L, Wang J, Zhou Y. A sub-millinewton resolution biaxial force sensor with temperature self-compensation for vascular intervention. Sensors Actuators A Phys. 2023;364: Article 114833.

[55] Li T, Shi C, Ren H. Three-dimensional catheter distal force sensing for cardiac ablation based on fiber Bragg grating. IEEE/ASME Trans Mechatron. 2018;23(5):2316–2327.

[56] Zhang T, Chen B, Zuo S. A novel 3-DOF force sensing microneedle with integrated fiber Bragg grating for microsurgery. IEEE Trans Ind Electron. 2021;69(1):940–949.

[57] Liang Q, Ouyang S, Long J, Zhou L, Zhang D. A novel fiber Bragg grating three dimensional force sensor for medical robotics. IEEE/ASME Trans Mechatron. 2024.

