# Supplementary material for: Multifunctional Magnetic Catheter Robot with Triaxial Force Sensing Capability for Minimally Invasive Surgery
Source: Research (Wash D C). 2025 Apr 24;8:0681. doi: 10.34133/research.0681 (PMC12018763; doi:10.34133/research.0681)
Supplement: Supplementary 1 — Figs. S1 to S7 Tables S1 and S2 Movies S1 to S3 [file research.0681.f1.zip › Research_Supplementary Materials_R3 for Proof.docx]

Supplementary Materials for

**“Multifunctional Magnetic Catheter Robot with Triaxial Force Sensing Capability for Minimally Invasive Surgery”**

Shixiong Fu, Shiyuan Dong, Haolan Shen, Zhiqiang Chen, Guoyao Ma, Mingxue Cai, Chenyang Huang, Qianbi Peng, Chenyao Bai, Yuming Dong, Huanhuan Liu,

Tianyu Yang*, and Tiantian Xu*

*Corresponding author: ty.yang@siat.ac.cn, tt.xu@siat.ac.cn

**This file includes:**

Fig. S1 to S7

Tables S1 and S2

Legends for movies S1 to S3

**Other Supplementary Material for this manuscript includes the following:**

Movies S1 to S3

**Supplementary Figures**


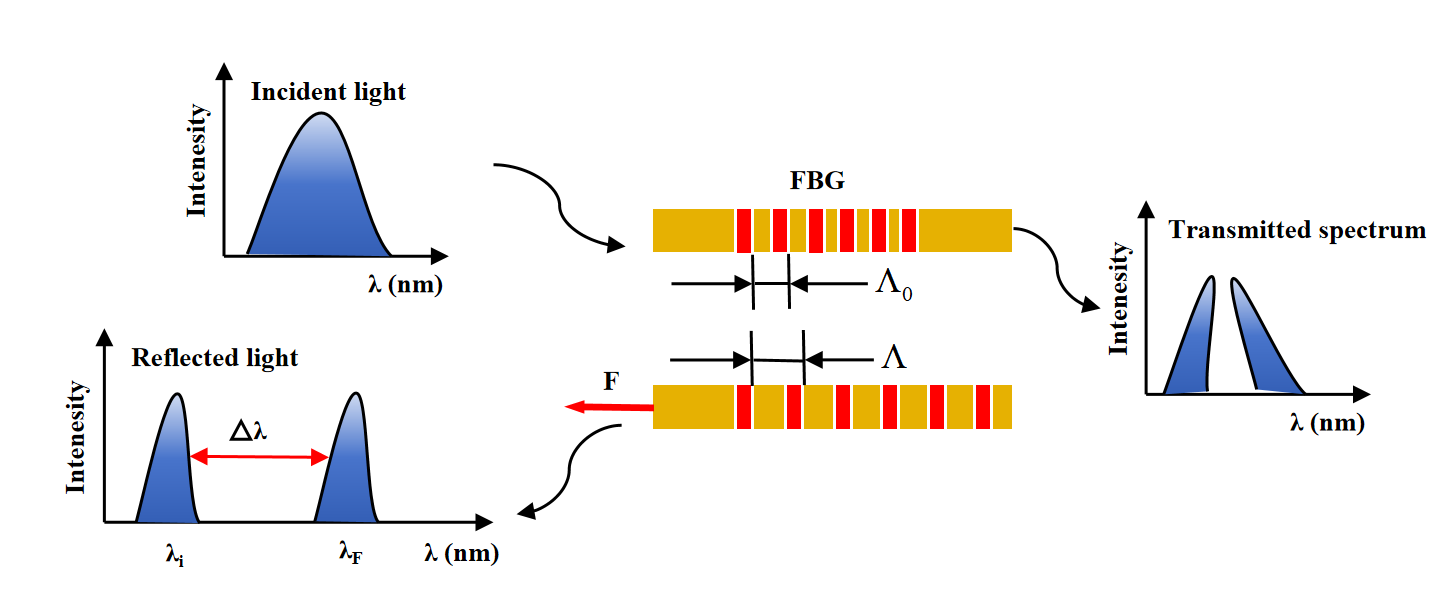


**Fig. S1.** Schematic diagram of the FBG for force sensing. When incident light passes through the FBG, a specific wavelength (Bragg wavelength) is selectively reflected, while other wavelengths are transmitted. The grating period, denoted as , determines the Bragg wavelength. When an external force F is applied, the grating period changes to , causing a shift in the reflected wavelength. This wavelength shift is directly proportional to the applied force or strain, enabling FBG to function as a highly sensitive sensor for stress or strain variations.

**
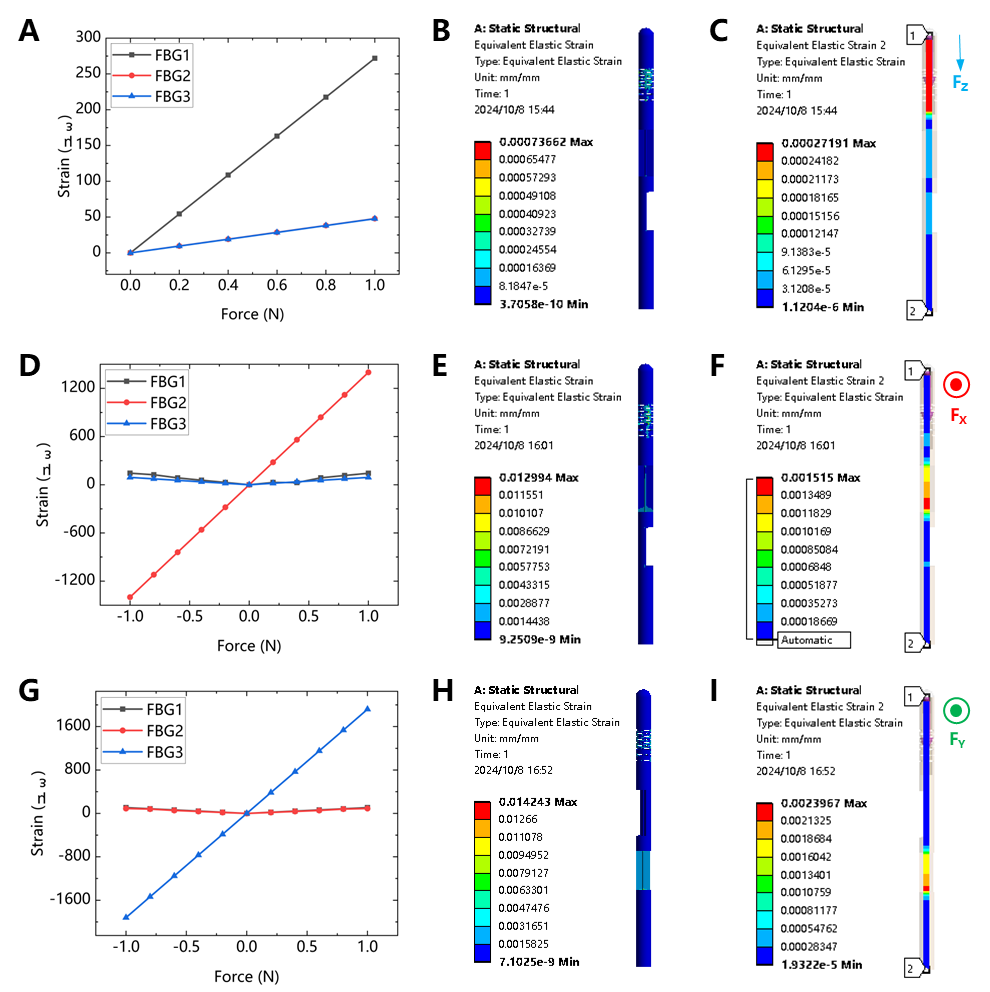
**

**Fig. S2.** Finite element simulation results of the force sensor in the three-axis directions. (A) The strain of the force sensor when the sensor applies different Z-direction axial forces. (B)-(C) The strain of the sensor and the strain of the optical fiber when an axial force of 1 N is applied. (D) The strain of the force sensor when the sensor applies different X-direction lateral forces. (E)-(F) The strain of the sensor and the strain of the optical fiber when a 1-N X-direction lateral force is applied. (G) The strain of the force sensor when the sensor applies different Y-direction lateral forces. (H)-(I) The strain of the sensor and the strain of the optical fiber when a 1-N Y-direction lateral force is applied.


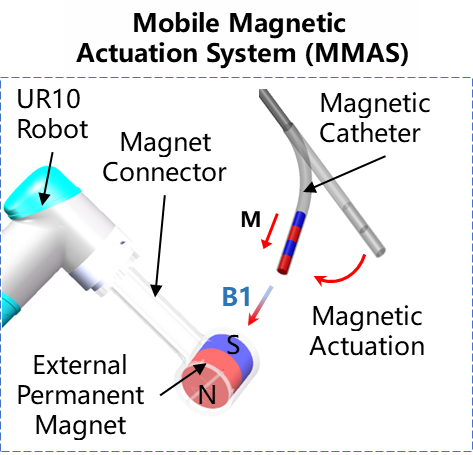


**Fig. S3.** Structure and working principle of mobile magnetic actuation system. The MMAS consists of a UR10 robotic arm and external permanent magnets. An axially magnetized N52-grade cylindrical NdFeB permanent magnet (D × L: 60 × 60 mm) is mounted to the UR10 robot as the end-effector through a 3D printed magnet connector. We control the end motion of UR10 to change the pose of the permanent magnet so that the magnetic catheter can be deflected to the desired state under the actuation of an external magnetic field.


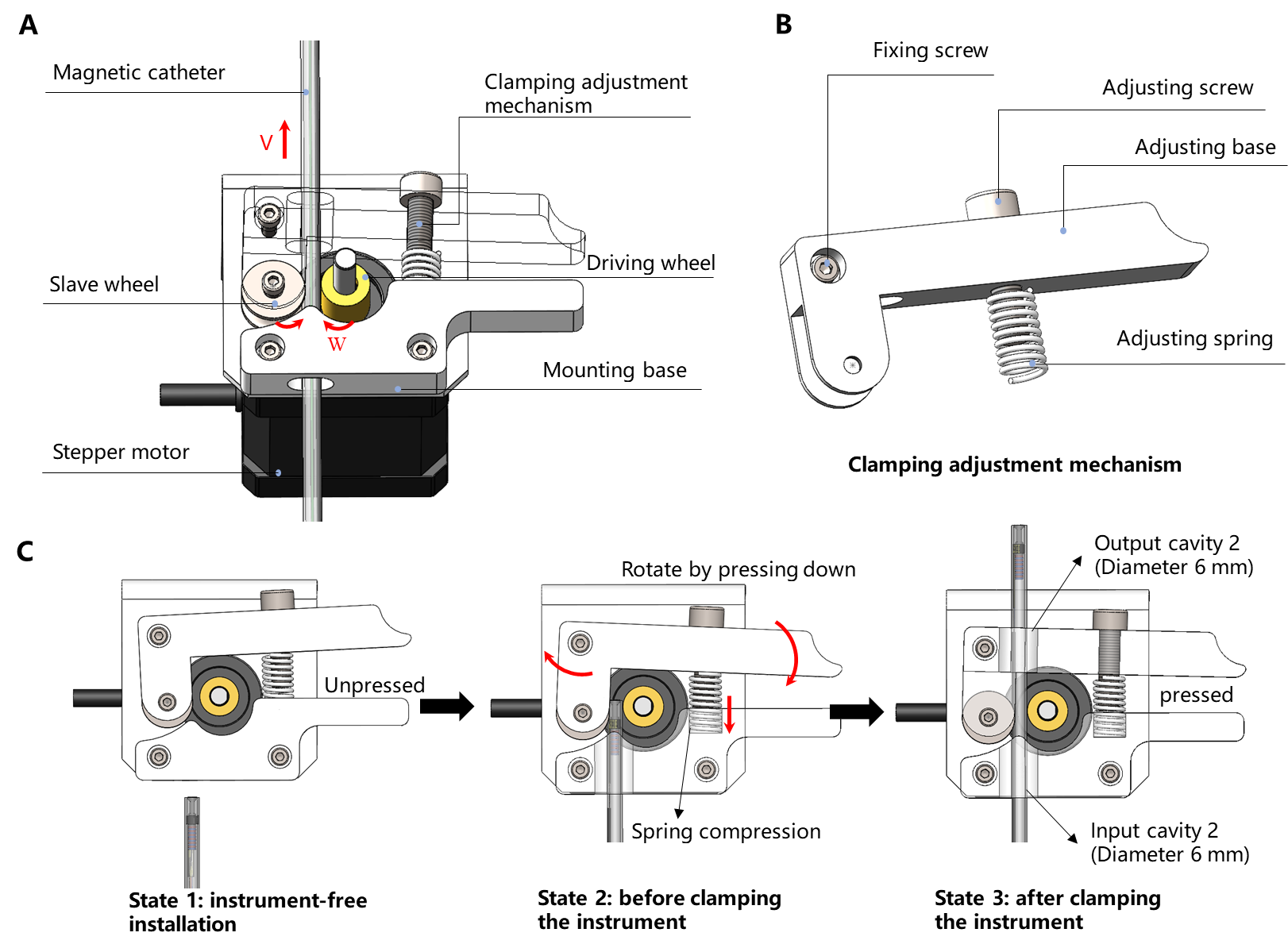


**Fig. S4.** The linear propulsion module and the instrument installation process of the catheter advancer (CA). (A) The detailed structure illustration of the catheter advancer. (B) The detailed compositional structure of the clamping adjustment mechanism in (A). (C) The instrument installation process and the state comparison of the before/after clamping the instrument. The red arrows in the figure indicate the direction of component motion and the direction of the external magnetic field.


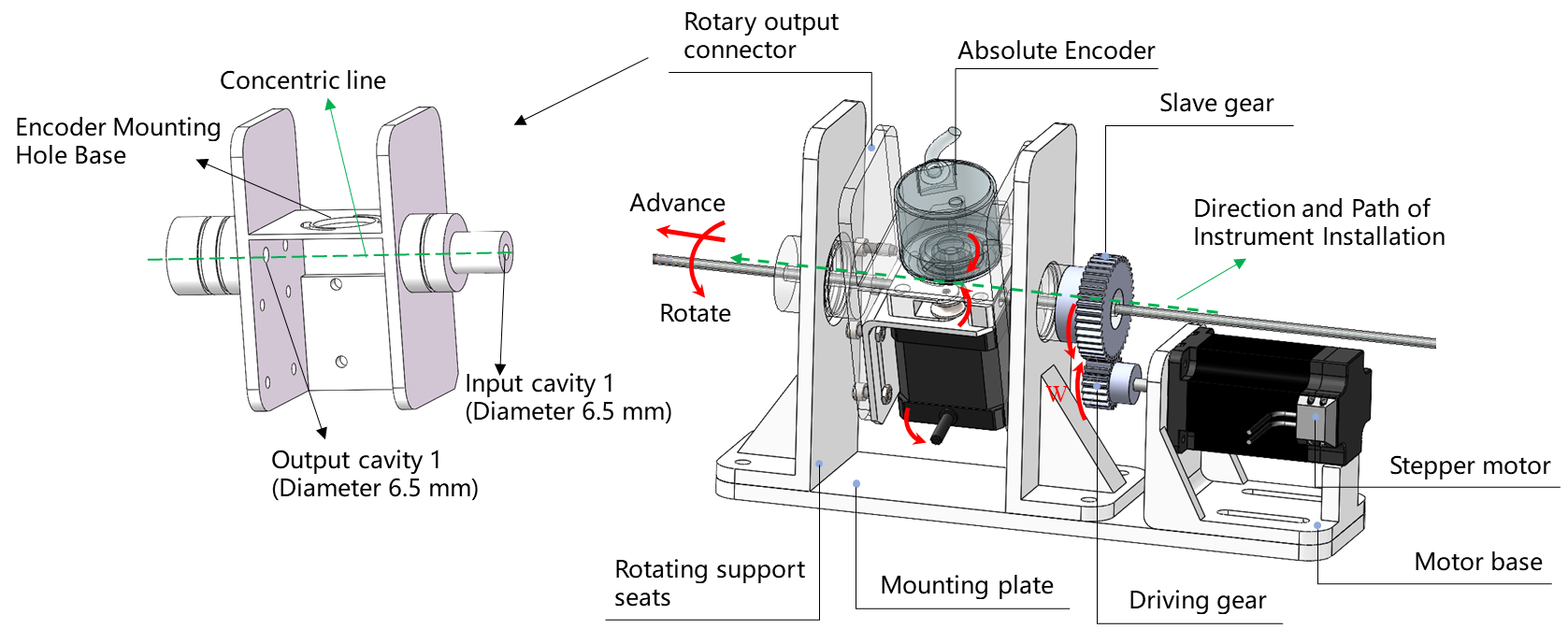


**Fig. S5.** Diagram of the structural components of the rotary motion module and the operating schematic of simultaneous linear and rotary motion of the catheter advancer (CA). The red arrow in the right figure indicates the direction of motion, and the green dotted line indicates the direction and path of the instrument installation. The left figure indicates the structure of the rotary output connector, and the green dashed line indicates the concentric line.


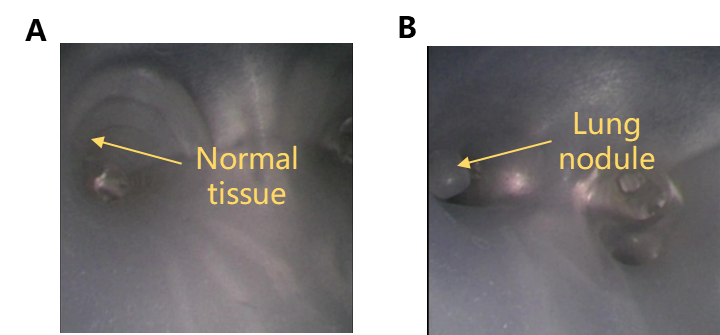


**Fig. S6**. Comparison of endoscopic imaging and tissue differentiation using the proposed magnetic catheter. (A) Endoscopic images of normal tissue. (B) Endoscopic images of lung nodule.


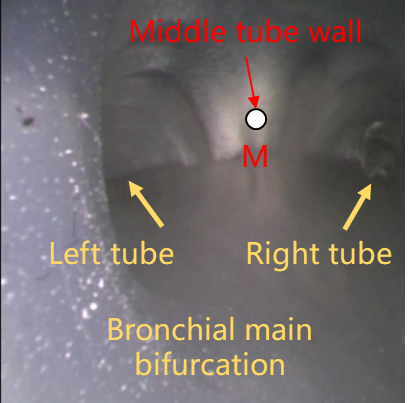


**Fig. S7**. Endoscopic image of the left and right tube and the middle tube wall at the bronchial main bifurcation.

**Supplementary Tables**

**Table S1. Detailed material parameters of the FBG sensor**

| Specification | Elastomer | Optical fiber |
| --- | --- | --- |
| Material | 304 stainless steel | Silica |
| Young's modulus (GPa) | 195 | 72 |
| Poisson's ratio | 0.3 | 0.17 |
| Density (kg/m3) | 7980 | 2500 |

**Table S2. Comparison of performance of FBG triaxial force sensors**

| References | Axles | Fibers | Diameter | Length | Resolution  (X&Y&Z) (mN) | Applications |
| --- | --- | --- | --- | --- | --- | --- |
| Lv[1] | Single | 1 | 5 mm | 19 mm | 2.55 | Palpation |
| Deng[2] | Single | 1 | 2 mm | 12.5 mm | 100 | None |
| Shi[3] | Double | 4 | 4 mm | 18 mm | 4.6 | None |
| Gan[4] | Double | 5 | 3.5 mm | 20 mm | 0.761&0.765 | Vascular intervention |
| Gao[5] | Triaxial | 4 | 2.2 mm | 11 mm | 2&1.8&1.3 | None |
| Gan[6] | Triaxial | 5 | 3.5 mm | 21.25 mm | 1.65&2.66&7.66 | Catheter ablation |
| Dong[7] | Triaxial | 3 | 3 mm | 15.5 mm | 4.3&3.99&3.3 | Palpation |
| Li[8] | Triaxial | 5 | 4 mm | 28 mm | 2.13&2.52&23.12 | Palpation |
| **This work** | **Triaxial** | **1** | **1 mm** | **18 mm** | **0.75&1.6&2.02** | **Palpation:**  **(Lung nodules in bronchial,**  **Polyps in stomach)** |

**References:**

[1] C Lv, S Wang and C Shi, "A high-precision and miniature fiber Bragg grating-based force sensor for tissue palpation during minimally invasive surgery," in Annals of biomedical engineering, 2020, 48: 669-681.

[2] Y Deng, T Yang, B Lou, et al, "A sensitivity difference approach to overcome temperature influence on a fiber optic force sensor with a pair of FBGs," in Sensors and Actuators A: Physical, 2022, 342: 113645.

[3] C. Shi, T. Li and H. Ren, "A Millinewton Resolution Fiber Bragg Grating-Based Catheter Two-Dimensional Distal Force Sensor for Cardiac Catheterization," in IEEE Sensors Journal, vol. 18, no. 4, pp. 1539-1546, 15 Feb.15, 2018, doi: 10.1109/JSEN.2017.2779153.

[4] L Gan, J Wang and Y Zhou, "A sub-millinewton resolution biaxial force sensor with temperature self-compensation for vascular intervention," in Sensors and Actuators A: Physical, 2023, 364: 114833.

[5] A. Gao, Y. Zhou, L. Cao, Z. Wang and H. Liu, "Fiber Bragg Grating-Based Triaxial Force Sensor With Parallel Flexure Hinges," in IEEE Transactions on Industrial Electronics, vol. 65, no. 10, pp. 8215-8223, Oct. 2018, doi: 10.1109/TIE.2018.2798569.

[6] L. Gan, J. Wang, L. Xie and Y. Zhou, "A High Precision Triaxial Force Sensor Based on Fiber Bragg Gratings for Catheter Ablation," in IEEE Transactions on Instrumentation and Measurement, vol. 73, pp. 1-11, 2024, Art no. 7001511, doi: 10.1109/TIM.2023.3342245.

[7] S. Dong et al., "A High-Precision Miniature Triaxial FBG Force Sensor for Detecting Tissue Anomalies," in Journal of Lightwave Technology, vol. 42, no. 17, pp. 6143-6152, 1 Sept.1, 2024, doi: 10.1109/JLT.2024.3403206.

[8] T. Li, C. Shi and H. Ren, "Three-Dimensional Catheter Distal Force Sensing for Cardiac

Ablation Based on Fiber Bragg Grating," in IEEE/ASME Transactions on Mechatronics, vol.

23, no. 5, pp. 2316-2327, Oct. 2018, doi: 10.1109/TMECH.2018.2867472.

**Supplementary Movies**

**Movie S1.**

Ex vivo Palpation of Simulated Tumor Lumps in the Porcine Kidney.

**Movie S2.**

Endoscope-assisted Palpation of Lung Nodules in Bronchial Phantom.

**Movie S3.**

Palpation and Biopsy of Simulated Polyps in Stomach Phantom.
